# Non-Adherence to Anti-Osteoporosis Medication: Factors Influencing and Strategies to Overcome It. A Narrative Review

**DOI:** 10.3390/jcm12010014

**Published:** 2022-12-20

**Authors:** Giulia Rita Agata Mangano, Marianna Avola, Chiara Blatti, Alessia Caldaci, Marco Sapienza, Rita Chiaramonte, Michele Vecchio, Vito Pavone, Gianluca Testa

**Affiliations:** 1 Department of Biomedical and Biotechnological Sciences, Section of Pharmacology, University Hospital Policlinico-San Marco, University of Catania, 95123 Catania, Italy; 2 Department of General Surgery and Medical Surgical Specialties, Section of Orthopaedics and Traumatology, University Hospital Policlinico-San Marco, University of Catania, 95123 Catania, Italy

**Keywords:** osteoporosis, adherence medications, elderly, frailty, postmenopausal, fractures, post-fracture care, educational meeting, drug regimen, medication monitoring

## Abstract

To evaluate the reasons for inadequate adherence to osteoporosis therapy and to describe the strategies for improving adherence to and persistence with regular medications, we conducted a review of the literature. The primary outcome of the study was the determination of the factors adverse to the onset and maintenance of anti-osteoporosis therapies. Secondly, we focused on studies whose efforts led to finding different strategies to improve adherence and persistence. We identified a total of 26 articles. The most recurrent and significant factors identified were aging, polypharmacy, and smoking habits. Different strategies to guide patients in their osteoporosis care have been identified, such as monitoring and follow-up via telephone calls, email, and promotional meetings, and proactive care interventions such as medication monitoring, post-fracture care programs, and decision aids. Changes in the drugs regimen and dispensation are strategies tried to lead to better adherence and persistence, but also improved satisfaction of patients undergoing anti-osteoporosis treatment. Patient involvement is an important factor to increase medication persistence while using a flexible drugs regimen.

## 1. Introduction

Osteoporosis is one of the main health problems, affecting more than 200 million people worldwide [1] and a major public health issue considering that fragility fractures, one of the most serious complications, result in significant increases in morbidity and mortality, as well as socioeconomic burden [2].

Safe and effective medications are available to reduce the risk of fractures, but numerous patients do not start or do not appropriately follow treatment for osteoporosis, leading to a significant clinical and financial burden [3,4].

The utilization of health services in a general way is determined by the interaction between predisposing factors (e.g., race, age, and health beliefs), enabling factors (e.g., social support and access to health services), and the perceived and actual need for healthcare services [5].

The process by which patients take their medications as prescribed is defined by the term adherence and includes initiation, implementation, and discontinuation. Terms such us compliance and persistence are used in the literature as well [6].

Initiation, implementation, and persistence with osteoporosis medications, especially oral bisphosphonates, but also other medications such us teriparatide, raloxifene, denosumab, and zoledronic acid, are proven to be suboptimal by several studies [7]. For instance, after medical prescription, about 20–30% of patients do not initiate taking oral bisphosphonates, [7] and the persistence rates at 1 year are commonly estimated between 16 and 60% [8]. Numerous and multidimensional reasons and factors influence nonadherence, varying for each patient [9].

This trend of poor persistence and adherence to osteoporosis medications lowers the gains in bone mineral density (BMD), which is the protecting factor against fragility fractures [10].

Furthermore, investing the interventions to increase drug adherence could improve health outcomes and the efficiency of the health system [11]. Economic studies have also suggested that improving adherence results in cost-effectiveness benefits [12]. Improving medication adherence could lead to greater benefits than designing a new, more effective drug [6].

Therefore, improving adherence to osteoporosis medications remains a pivotal, but challenging task. Several interventions and programs have therefore been developed to improve osteoporosis medications adherence [3].

The primary outcome of the study was the determination of the factors adverse to the onset and maintenance of anti-osteoporosis therapies. Secondly, we focused on studies whose efforts led to finding different strategies to improve adherence and persistence.

## 2. Materials and Methods

We performed an extensive literature search in PubMed, Scopus, and Web of Science to identify relevant studies published from January 2012 to January 2022, analyzing the factors influencing osteoporosis medication adherence and interventions to improve it. The following search was used: (Osteoporosis AND (compliance OR adherence)) AND (prevention OR medications for treatment of therapy or follow up or exam or diagnosis). We limited our search to English language publications. A total of 308 studies were found. Upon reviewing the titles, excluding those articles which did not examine the barriers to starting or continuing anti-osteoporosis intervention and did not study the methods to ameliorate it, we identified a total of 26 articles.

## 3. Results

We identified 8 studies exploring the barriers and factors influencing patients’ adherence, 10 studies testing and analyzing methods (healthcare systems, interventions, programs) to improve medication adherence, and 8 more studies which had as a main focus changes in administration methods as a strategy to increase persistence. The characteristics of the studies, samples, outcome measures and/or results are set out in Table 1, Table 2 and Table 3.

### 3.1. Factors and Barriers Influencing Adherence and Persistence

The most frequent factors influencing anti-osteoporosis medication adherence or persistence identified are aging, polypharmacy, and smoking habits. Alamri et al.’s study, [15] in 2015, found that the Clinical Practice Guidelines for the Diagnosis and Management of Osteoporosis in Canada, [37] are underutilized in long-term care (LTC).

The most commonly reported barriers to providing optimal bone health care in LTC were a lack of information and educational resources, difficulty obtaining the required patient information for a fracture risk assessment and the inconsistent prescribing of vitamin D and calcium at the time of admission, as well as the difficulty in including osteoporosis and fracture prevention strategies as topics for quarterly reviews.

In 2017 Fahrleitner-Pammer conducted a study assessing persistence, adherence, and medication coverage ratio (MCR) in postmenopausal women receiving denosumab in routine practice in Germany, Austria, Greece, and Belgium [13]. Lower persistence was associated with elderly age, a history of previous interruptions in therapy, patients’ intolerance to other osteoporosis medications, smoking habit, and a history of falls in the year before enrollment. Individuals with multiple comorbidities are likely to have a high medication burden [38] which may be confusing and could result in osteoporosis treatment being considered a low priority. In addition, when attempting to reduce the medication burden, physicians and patients may deprioritize osteoporosis therapy.

In García-Sempere’s study, aging was associated with both non-adherence and non-persistence, similar to what has been seen previously [14]. Poor adherence-only was associated with sedative treatment and previous stroke, while being male and having dementia led mostly to impaired persistence. A high medication burden, due to multiple comorbidities, led to lower non-adherence in this study as well.

Aging, comorbidities, and smoking attitude were identified as worsening factors for adherence to osteoporosis therapy, probably because these conditions could express the reduced attention of the patients to their state of health as was explained in the randomized prospective study conducted by Gonnelli et al. in 2016 [16]. Moreover, being overweight was associated with worse compliance too. The authors explained this finding with a likely reduced attention of the patients towards their health condition. They tried to ameliorate compliance through patient information about fracture risk using the DeFRA algorithm [39], but this strategy only marginally improved adherence. Osteoporosis prior to fracture is asymptomatic, and patients are more likely to prioritize other diseases that have a more direct impact on their daily lives.

On the other hand, in the study conducted by Parsons et al., [19] the use of systematic screening for fracture risk using FRAX^®^ in primary care led to the increased use of, and adherence to, anti-osteoporosis medications, compared to usual care.

Hall et al. examined the effects of a patient-activation intervention on osteoporosis pharmacotherapy through osteoporosis knowledge, conducting interviews about the reasons for non-adherence to therapy [17]. The most common reasons for non-adherence were the fear of side effects or contraindications, a dislike of taking medications, and believing that the prescribed medication would not improve their condition.

McAlister et al. analyzed the effect of an educational program, comparing it with usual care from a primary care physician through an RCT, identifying the factors influencing compliance to biphosphonates [18]. The most commonly reported reasons for stopping bisphosphonate therapy were the side effects, mostly gastrointestinal ones. Moreover, this study highlights the importance of established physician–patient relationships and continuity of care in the decision to take long-term preventive therapies. In fact, patients who were managed by their physician, had a better 2-year adherence than patients dealt with by the educational program.

A similar scenario is encountered in the Salter et al. trial [20], in which it is suggested that preventive health measures often pose a challenge in which the general practitioner has to make individual decisions dependent on the beliefs, understanding, needs, and expectations of the patient in front of them, debating every new health issue in the context of the person’s whole life, maximizing health gain, and minimizing adverse consequences [40].

### 3.2. Methods and Healthcare Systems to Enhance Adherence and Persistence

Different strategies to guide patients in their osteoporosis care have been identified, such as monitoring and follow-up via telephone calls, email and education meetings, proactive care interventions, such as medication monitoring, post-fracture care programs, and decision aids.

#### 3.2.1. Telephone Calls, Emails, Educational Meetings

In an RCT, Tuzun et al. randomized patients with postmenopausal osteoporosis undergoing treatment with weekly oral bisphosphonates into two groups, an active training group (AT) and a passive training group (PT). Both groups received a Starter Training Kit including a bisphosphonate guide and osteoporosis training booklets. The AT group was, in addition, trained through a standard training package including telephone calls and interactive educational meetings. The authors evaluated persistency, treatment compliance, adverse events, vertebral and non-vertebral fractures, and quality of life. There were no significant differences between the AT and PT groups, most of the patients always used their drugs regularly according to the recommended days and dosages; the most common reason for not receiving treatment regularly was forgetfulness and most of the patients were highly satisfied with the treatment and wanted to continue [21].

In another Turkish study patients with post-menopausal osteoporosis undergoing treatment with weekly or monthly bisphosphonates were included and randomized into two groups. The training group was provided with a training kit including booklets containing information about osteoporosis and followed up with telephone calls and individual face-to-face interactive/educational meetings focused on disease awareness. The patients in the control group were followed up by physicians without supplying training booklets. The authors did not find significant differences between the training and control groups in terms of compliance and persistence. The patients on the monthly bisphosphonate regimen showed significantly longer persistence in comparison to patients on the weekly regimen [22].

In an RCT, Solomon et al. enrolled patients who had been newly prescribed a medication for osteoporosis and divided them into two groups. Both received information material on osteoporosis by e-mail, while the patients in the intervention group also received motivational interviewing counseling sessions via telephone with health educators discussing osteoporosis medication. They could not find a statistically significant improvement in adherence to an osteoporosis medication regimen using this method [23].

Post-menopausal women, receiving an oral prescription for osteoporosis for the first time were recruited and randomized into three groups in an RCT conducted by Bianchi et al. Group 1 were managed according to standard clinical practice, group 2 received educational booklets providing information on osteoporosis and reminders as well an alarm clock to prompt medication administration, group 3 also received phone calls from physicians and nurses who discussed the topic of osteoporosis with patients. The outcomes were adherence and persistence to therapy. There were no significant differences among the three groups. The authors point out that monthly intake of the drug had a higher adherence than weekly and daily intake [24].

Kessous et al. in an RCT investigated whether a clinical intervention after a distal radial fracture would encourage patients to visit their primary care physician and start an OP therapy. Seventy patients were divided into two groups. Both groups were contacted by telephone 6–8 weeks after the fracture and asked to respond to a questionnaire about their awareness of osteoporosis and fragility fractures. Only the intervention group received an explanatory pamphlet and an article about osteoporosis with a letter to their primary care physician that recommended further diagnostic workup. The outcome was evaluated by a second call for both groups after 6–8 weeks and was considered positive if the patients’ referral to their primary care physician had resulted in them undergoing an osteoporosis workup. The intervention increased the number of patients who turned to their primary care physician and boosted the proportion of patients undergoing a diagnostic examination [25].

#### 3.2.2. Medication Monitoring

In a study by Stuurman-Bieze the pharmacy provided structured counseling on aspects regarding administration, efficacy, and possible side-effects. The pharmacists checked whether the patients returned for their next prescriptions. If the patients did not redeem their medication they were contacted if warranted. The results were compared to a reference group receiving the usual pharmacy care. This intervention can decrease patients’ non-adherence; 93% of patients were satisfied and mentioned that the pharmacy was the only place where they received explanations regarding osteoporosis [26].

#### 3.2.3. Post-Fracture Care

Ganda et al. evaluated whether a secondary fracture prevention (SFP) program could improve compliance and persistence with oral bisphosphonate therapy. An intervention group was followed by a specialist in the SFP service for the entire duration of the study, while a control group was seen by the SFP service twice and then followed up by their primary care physician. At 24 months the medication persistence and medication possession ratio (MPR) were similar in both groups. Time-based changes in BMD or bone turnover were not associated with persistence and compliance. These results indicate that one of the main functions of an SFP program may be the initiation of therapy rather than continuous patient monitoring [27].

Merle et al. evaluated the impact of a population-based patient-centered post-fracture care program, PREVOST, in an RCT. The intervention group received a phone call where a trained case manager focused on the association between fragility fractures and the high risk of osteoporosis and encouraged the patient to visit their primary care physician to discuss their personal risk of fragility, fracture, and osteoporosis, to schedule a BMD test, and to start a pharmacological treatment for osteoporosis if necessary. An information summary booklet was then mailed to each subject. Reminder phone calls were performed following the telephone discussion. The patients from the control group received the usual care. The primary outcome was the percentage of patients who reported the beginning of appropriate post-fracture care. The secondary outcomes were the percentage of patients who reported that a BMD had been performed, a treatment prescription and/or a calcium–vitamin D supplementation had been given, and information on osteoporosis had been delivered by the primary care physician. The authors described a significantly improved post-fracture BMD investigation [28].

#### 3.2.4. Decision Aid

LeBlanc et al. enrolled women with a diagnosis of osteopenia or osteoporosis who were not taking medications to treat their condition and compared the patients’ estimated risk of fracture using the FRAX calculator with Osteoporosis Choice, an encounter decision aid. The latter included the individualized 10-year risk of having a bone fracture, using the FRAX calculator, with and without the use of bisphosphonate, and the possible harms and other disadvantage of using bisphosphonates. The primary outcomes were the patients’ decisional conflict, knowledge, decision whether to start medication, adherence to medication, involvement in decision making by the clinician, fidelity to the intended intervention, acceptability, satisfaction, and quality of life. The secondary outcome was decision quality. The Osteoporosis Choice decision aid was found to be better than usual care with or without the FRAX calculation. More patients started taking a bisphosphonate and filled their prescriptions in the decision aid group arm compared to the FRAX/usual care group. The FRAX calculator alone as a clinical decision support tool during the encounter was no different from usual care across all the measured parameters [41].

In a pilot randomized trial the authors tested the feasibility of a fracture prevention decision aid in an online patient portal. The patients in the intervention group received the decision aid which contained a 10-year fracture risk calculator, a summary of the medication risks and benefits (prescription and nonprescription), and an elicitation of values, while those in the control group were directed to the National Institute on Aging homepage which provided web-based information relevant to aging but not specific to osteoporosis. The first outcomes were decisional conflict and preparation for decision making; the second outcomes were feasibility and planning for a larger trial. The patients in the intervention group reported being more prepared for making decisions about their treatment and having decreased decisional conflict compared to the patients in the control group [42].

### 3.3. Drug Regimen

Changes in drug regimen and dispensation are strategies attempted to lead not only to better adherence and persistence, but also improved satisfaction of patients undergoing treatment with anti-osteoporosis medication. Various studies explore this issue.

Oral et al. conducted a multicentric study including women with post-menopausal osteoporosis (OP) [29]. They evaluated the level of compliance and persistence over 26 weeks in women receiving risedronate daily, following two different regimens: flexible doses or fixed doses either before breakfast, in-between meals or before bedtime. In both groups the effect on the urinary N-terminal telopeptide of type 1 collagen was evaluated. The study resulted in a higher rate of persistence among patients under the flexible regimen; however, no statistical significance was noted in terms of compliance between the two groups.

Finigan et al. analyzed adherence to raloxifene for 2 years among post-menopausal women [30]. They compared the methods of tablet counts and of electronic monitoring with electronic bottle caps and eventually they examined the degree of bone response to raloxifene. Simple counts of returned tablets may mask irregular patterns of tablet-taking, therefore electronic monitoring is the most accurate way to monitor actual behavior and the resulting adherence levels are consistently lower than those obtained by counting returned tablets.

Freemantle et al. conducted a study that compared adherence, compliance, and persistence in a group of post-menopausal osteoporotic women who were firstly administered once-weekly alendronate tablets for 12 months and then for another 12 months were administered subcutaneous denosumab injections every 6 months; the other group followed the opposite pattern of osteoporosis therapy medication [31]. Denosumab was associated with less non-adherence than alendronate. Postmenopausal osteoporotic women were shown to be more adherent, compliant, and persistent with subcutaneous denosumab injections every 6 months than with once-weekly alendronate tablets. These results are aligned to the degree of satisfaction: women preferred injectable denosumab over oral alendronate. The BMD variation was analyzed, and further improvements were described when subjects received alendronate first followed by denosumab, with BMD after the opposite pattern remaining stable.

In addition, Kendler et al. conducted a study of a group of post-menopausal osteoporotic women who during the first year were administered 70 mg of alendronate daily, and during the second year were given a subcutaneous denosumab injection every 6 months [32]. At baseline, 6, 12, 18, and 24 months, patients answered a questionnaire about the necessity of treatment and their concerns regarding osteoporosis therapy. The BMQ (Beliefs about Medicines Questionnaire) results showed that the subjects included in the study reported a greater preference for denosumab to alendronate in both treatment periods.

Muratore et al. conducted a 3-year randomized study with post-menopausal women affected by rheumatic arthritis and glucocorticoid induced osteopenia (T score ≥ 2.5) [33]. A total of 87 patients receiving methylprednisolone therapy were randomized into three treatment pattern groups for 1 year: 30 on neridronate, 27 on alendronate, and 30 on risedronate. They compared the adherence to intramuscular neridronate versus oral alendronate or risedronate therapy. The results from the study showed a higher adherence to intramuscular neridronate administered monthly than oral alendronate or risedronate administered weekly. Neridronate was shown to have similar efficacy to alendronate or risendronate in terms of BMD.

Palacios et al. considered the TSQM (Treatment Satisfaction Questionnaire for Medication) for the first time for the evaluation of osteoporosis treatment satisfaction [34]. They enrolled in the study post-menopausal osteoporotic women that had already been undergoing osteoporotic therapy for at least 1 month prior to screening with daily or weekly oral alendronate for transition to risedronate, and any oral bisphosphonate for transition to ibandronate. The patients were randomized to be administered subcutaneous denosumab every 6 months, or oral ibandronate or risedronate monthly for 12 months. The study showed that women with post-menopausal osteoporosis were more satisfied after transitioning to subcutaneous denosumab every 6 months compared with transitioning to risedronate or ibandronate every month.

Roh et al. conducted a study of women with distal radius fractures and limited health literacy [35]. These patients were randomized into two groups: one underwent intravenous ibandronate injections every 3 months for 1 year and the other group were administered weekly alendronate orally for 1 year. They reported a higher adherence in the subjects receiving intravenous ibandronate injections treatment than those receiving alendronate per os every 3 months, justified both by the pattern of administration and by the gastrointestinal adverse events.

Tamechika et al. conducted a study with patients affected by glucocorticoids induced osteoporosis, treated with weekly alendronate or risedronate [36]. These patients were randomized into two groups: one group continued their original bisphosphonate treatment weekly, the other group switched to monthly minodronate. Satisfaction therapy and BMD at the lumbar spine level were evaluated. Even though drug compliance in both groups was excellent and not statistically significant, switching to monthly minodronate considerably improved patient satisfaction as well as decreasing TRACP-5b (a bone resorption marker) and increasing BMD; however, serum BAP level (a bone formation marker) showed no significant difference between the two groups.

## 4. Discussion

In the literature it is well established that osteoporosis treatment considerably decreases the risk of non-vertebral and vertebral fractures. The management of osteoporosis is arduous since patients with osteoporosis can be totally asymptomatic until they have a fracture, contrarily to other chronic pathologies such as diabetes or heart failure. Due to poor adherence to osteoporosis treatment, patients develop poor clinical conditions. Therefore, the need to improve this situation is one of the most important issues in the treatment of osteoporosis. Improving adherence to osteoporosis medications is a challenging task. The reasons for nonadherence to osteoporosis treatment are several and multifaceted and differ for each patient [9].

It is clear that the current usual practice regarding the assessment of osteoporosis after a fragility fracture is insufficient. There are no appropriate guidelines regarding the correct follow-up and treatment of patients with fragility fractures; furthermore, a fragility fracture patient is seen by numerous doctors who may lack the required lines of communication with one another. Usually patients with a femoral neck fracture tend to experience a long hospital placement and for this reason they are more likely to receive an explanation concerning the association between their fracture and osteoporosis.

Various interventions and programs have been designed to improve osteoporosis medications adherence. Most of the studies evaluating adherence to and persistence in osteoporosis treatment are based on patient education, using different methods. The use of telephone calls, emails, and alarm clocks as a reminder to take medication as prescribed compared with the usual care pathways seems not to improve adherence and persistence [21,22,23,24,25].

Explanatory pamphlets, articles, and training booklets regarding the correlation between fragility fractures and osteoporosis can increase awareness and consequently the percentage of patients who start a diagnostic examination pattern for osteoporosis, as well as increasing the number of patients who turned to their primary care physician. In the studies evaluated there was a significant difference in patient participation and involvement. If the patient was advised about and involved in the therapeutic prescription decision regarding their drug regimen, there was an improvement in continuation; conversely there was no improvement in adherence when the patient was not involved [26]. Well-informed patients seem to take their medication regularly [21]. Patient involvement is an important factor to increase medication persistence while using a flexible dosing regimen. Coherent with the concept that reducing the complexity and frequency of dosing regimens improves adherence to and persistence with bisphosphonates in patients with osteoporosis, several authors point out that a monthly intake of osteoporosis medications has a higher adherence and persistence in comparison to patients on weekly and daily regimens [22,24]. Switching from a weekly to a monthly bisphosphonate regimen seems to offer a helpful strategy for improving long-term fracture prevention [22]. It seems that changing the drug regimen is only helpful for patients already using osteoporosis drugs and not for new medication treatments [43].

Other interventions to increase adherence and persistence include Medication Monitoring and Optimization, where pharmacies provided structured counseling on aspects regarding the administration, efficacy, and possible side-effects of medications, in order to reduce the fear of therapy and encourage take-up, showing that this decision aid or better-than-usual care decreases decisional conflict and increases patient knowledge and involvement in deciding to start therapy [41,42]. Decision aid communicates not only the risk of fracture but also quantifies the potential risk reduction with bisphosphonate therapy. Decision aid also brings various essential patient topics (i.e., side effects, cost) to the forefront and serves as an invitation for the patient and clinician to address these [41]. Patient portal-based decision aid was also effective at decreasing decisional conflict, preparing patients to make a decision on how to prevent fractures, and increasing patients’ self-reported decision making [42].

Secondary fracture prevention programs should identify patients and initiate treatment rather than facilitate continuous patient management. These programs not only overcome the aversion to initiating the appropriate management of patients with incident osteoporotic fractures, but also result in high compliance and persistence with treatment over time [27]. Some authors have suggested that the initiation of bisphosphonate therapy soon after an incident fracture may improve compliance and persistence because the acute fracture event provides a window of opportunity to instigate positive behavioral change [44].

Moreover, understanding patients’ perceptions and preferences for treatment may be an effective method for improving adherence to the appropriate osteoporosis therapy selected [32]. Medications with longer intervals between doses and a reduced risk of gastrointestinal issues, such as neridronate, minodronate, or denosumab compared with a weekly intake of bisphosphonate, are proven to increase patients’ satisfaction and therefore compliance [33,34].

## 5. Conclusions

This review tried to explore the limitations, barriers, and factors influencing anti-osteoporosis medication adherence, finding that generally patient education, monitoring, changes in drug regimens combined with patient support, and patient education through interdisciplinary collaboration has been shown to have positive effects on adherence to and persistence with treatment. Greater treatment satisfaction may lead to better treatment adherence and, ultimately, improvements in treatment effectiveness.

## Figures and Tables

**Table 1:** Characteristics of studies analyzing factors and barriers influencing adherence and persistence.

Author	Study Design	Sample Size	Outcome Measures	Results/Conclusions
Fahrleitner-Pammer et al., 2017 [13]	Multicenter, prospective, noninterventional study	1500	24 months Denosumab persistance. Medical CRatio and Morisky Medication Adherence Scale (MMAS-8) questionnaire	Falling episodes before enrolling, multiple comobridities, age > 75 years, smoking habit
Garcia-Sempere et al., 2017 [14]	Population-based retrospective cohort	4856	Primary and secondary non-adherence (proportion of days covered (PDC) and persistence) to osteoporosis medications 1 year and 4 years after the first prescription	Age, dementia, polypharmacy, previous diagnosis of osteoporosis, rheumatoid arthritis
Alamri et al., 2015 [15]	Qualitative study	40	Interviews about barriers to implementing osteoporosis and fracture prevention guidelines	Lack of information and educational resources, difficulty obtaining required patient information for fracture risk assessment, inconsistent prescribing of vitamin D and calcium at the time of admission
Gonnelli et al., 2016 [16]	Retrospective and prospective study	3206 + 816	4-item Morisky Medication Adherence Scale (MMAS)	History of osteoporotic fractures, frequency of drug administration, condition of being overweight/obese, age, smoking habit
Hall et al., 2017 [17]	Randomized controlled trial	790	Patient information and knowledge of osteoporosis through interview, FRAX	Osteoporosis knowledge, fear of medicine
McAlister et al., 2018 [18]	Randomized controlled trial	129	2 months’ adherence (>80% of dose assumed), rate of non-adherence, 24 months’ adherence to biphosphonates through self-report and pills report, SF-12, DASH, OptQol	Family members with osteoporosis, physician–patient relation
Parsons et al., 2019 [19]	Exploratory study	12,483	Self-reported adherence questionnaire	Incident fracture, prior medication, age, screening for fracture risk using FRAX
Salter et al., 2014 [20]	Longitudinal qualitative study	30	Interviews about understanding of osteoporosis, responses to screening results, current usage of preventive medicine, motivators and detractors from taking medication, follow-up with healthcare professionals	Severe side effects, confusion, lack of knowledge about the risks of osteoporosis

**Table 2:** Characteristics of studies analyzing methods and healthcare systems to enhance adherence and persistence.

Author	Study Design	Treatment	Sample Size	Outcome Measures	Results/Conclusions
Tüzün et al., 2013 [21]	Multicenter randomized controlled study	Use of bisphosphonate guide and osteoporosis training booklets. Intervention group received four phone calls and participated in four interactive social/training meetings held in groups of 10 patients.	448 aged 45–75 years with postmenopausal osteoporosis	1. Self-reported persistence and compliance with the treatment. 2. Quality of life of the patients assessed by the 41-item Quality of Life European Foundation for Osteoporosis (QUALEFFO-41) questionnaire.	No significant differences between AT and PT groups in both visit 1 and visit 5.
Akarırmak et al., 2016 [22]	Prospective non-interventional observational cohort registry study	Use of “Training Kit”, including four training booklets (“General Information on Osteoporosis”, “Osteoporosis and Exercise”, “Osteoporosis and Nutrition”, “Osteoporosis and Patient Rights”) During 12-month follow-up, four telephone calls and four individual face-to-face interactive/educational meetings.	979 mean age 63.2(7.2) with postmenopausal osteoporosis	1. Persistence and compliance. 2. Effect of bisphosphonate treatment on withdrawals from the study due to adverse event.	No significant difference in terms of compliance and persistence (79.4% of the patients).
Solomon et al., 2012 [23]	Randomized pilot study	Telephonic motivational interviewing intervention.	2087 with osteoporosis	1. Medication regimen adherence.	In an intention-to-treat analysis, median adherence was 49% in the intervention arm and 41% in the control arm.
Bianchi et al., 2015 [24]	Randomized prospective study	Booklets providing information on osteoporosis and the importance of adherence to treatment. Colored memo stickers for a calendar or diary. Alarm clock. Phone calls.	334 post-menopausal women	1. Adherence to therapy. 2. Persistence with therapy.	247 of 334 patients (74%) started the prescribed therapy.
Kessous et al., 2014 [25]	Prospective randomized l trial	Use of explanatory pamphlet, article concerning OP, and a letter addressed to primary care physician that recommended further diagnostic workup.	99 with distal fractures radius	Referral to their primary care physician and undergoing an OP workup.	Intervention increased the number of patients who turned to their primary care physician from 22.9% to 68.6% and boosted the proportion of patients undergoing a diagnostic examination from 14.3% to 40% (*p* < 0.001).
Stuurman-Bieze et al., 2014 [26]	Prospective intervention study	Medication. Monitoring and Optimization (MeMO) intervention, compared to usual pharmacy care.	937 with osteoporosis	1. Therapy discontinuation and nonadherence. 2. Patients’ satisfaction.	32.8% of patients in the usual care group initiating osteoporosis medication were nonadherent or discontinued, compared to 19.0% of patients in the intervention group (*p* < 0.001).
Ganda et al., 2014 [27]	Randomized controlled trial	Secondary-fracture prevention handled by specialists, compared with usual care.	102 men and women with osteoporotic fractures	1. Patient compliance and persistence.	At 24 months’ medication persistence the medication possession ratio was similar in both groups (64% versus 61%, respectively; *p* = 0.75)
Merle et al., 2017 [28]	Multicenter, randomized controlled trial	PREVOST population-based patient-centered post-fracture care program, compared to usual care.	436 women aged 50–85 years (fracture of the wrist or humerus)	1. Proportion of women who reported the initiation of an appropriate post-fracture care program. 2. Proportion of bone mineral density scans performed.	At 6 months, 53% of patients in the intervention group began a post-fracture care program versus 33% in the control group.

**Table 3:** Characteristics of Studies analyzing different drug regimens.

Author	Study Design	Sample Size	Outcome Measures	Results/Conclusions
Oral et al., 2015 [29]	Multicentric, prospective, crossover, randomized, parallel	448 postmenopausal women	Persistence and compliance	Patients on a flexible daily dose of risedronate are more compliant and persistent than patients on fixed regimens.
Finigan et al., 2013 [30]	Single-center, prospective, randomized	75 women	Adherence	Monitoring caps assesses adherence more accurately than tablet counts.
Freementle et al. 2012 [31]	Single-center, randomized, open-label, crossover	250 women	Adherence, compliance, persistence, and satisfaction	Patients were more adherent, compliant, persistent, and satisfied with subcutaneous denosumab injections every 6 months than with once-weekly alendronate tablets.
Kendler et al., 2014 [32]	Multicenter, randomized, open-label, crossover	250 women	BMQ (Beliefs about Medicines Questionnaire)	Participants preferred denosumab to alendronate while on treatment and had more positive perceptions of denosumab than alendronate. These perceptions were associated with better adherence.
Muratore et al., 2013 [33]	Randomized, open-label, parallel-group, single centre	87 women	Adherence	Neridronate is associated with higher adherence and a better effect on BMD compared to alendronate and risedronate.
Palacios et al., 2015 [34]	Single-center, retrospective, randomized, open-label	1703	TSQM (Treatment Satisfaction Questionnaire for Medication)	Patients were more satisfied when transitioned to denosumab versus switching to a monthly oral bisphosphonate.
Roh et al., 2018 [35]	Single-center, randomized	439	Adherence	Patients with limited health literacy showed better adherence to quarterly intravenous bisphosphonates compared to weekly oral bisphosphonates, similar to rates among patients with appropriate literacy.
Tamechika et al., 2018 [36]	Multicentric, randomized, prospective, open- label	130	Satisfaction, BMD	Patients were more satisfied with monthly minodronate compared to weekly alendronate or risedronate, it also showed an improvement in BMD.

## Data Availability

Non applicable.
